# In-season internal and external training load quantification of an elite European soccer team

**DOI:** 10.1371/journal.pone.0209393

**Published:** 2019-04-22

**Authors:** Rafael Oliveira, João P. Brito, Alexandre Martins, Bruno Mendes, Daniel A. Marinho, Ricardo Ferraz, Mário C. Marques

**Affiliations:** 1 Sports Science School of Rio Maior–Polytechnic Institute of Santarém, Rio Maior, Portugal; 2 Research Centre in Sport Sciences, Health Sciences and Human Development, Vila Real, Portugal; 3 Life Quality Research Centre, Santarém, Portugal; 4 Department of Sports Sciences, University of Beira Interior, Covilhã, Portugal; 5 Faculty of Human Kinetics, University of Lisbon, Lisbon, Portugal; 6 Football Association of Castelo Branco, Castelo Branco, Portugal; Instituto Politecnico de Viana do Castelo, PORTUGAL

## Abstract

Elite soccer teams that participate in European competitions need to have players in the best physical and psychological status possible to play matches. As a consequence of congestive schedule, controlling the training load (TL) and thus the level of effort and fatigue of players to reach higher performances during the matches is therefore critical. Therefore, the aim of the current study was to provide the first report of seasonal internal and external training load that included Hooper Index (HI) scores in elite soccer players during an in-season period. Nineteen elite soccer players were sampled, using global position system to collect total distance, high-speed distance (HSD) and average speed (AvS). It was also collected session rating of perceived exertion (s-RPE) and HI scores during the daily training sessions throughout the 2015–2016 in-season period. Data were analysed across ten mesocycles (M: 1 to 10) and collected according to the number of days prior to a one-match week. Total daily distance covered was higher at the start (M1 and M3) compared to the final mesocycle (M10) of the season. M1 (5589m) reached a greater distance than M5 (4473m) (ES = 9.33 [12.70, 5.95]) and M10 (4545m) (ES = 9.84 [13.39, 6.29]). M3 (5691m) reached a greater distance than M5 (ES = 9.07 [12.36, 5.78]), M7 (ES = 6.13 [8.48, 3.79]) and M10 (ES = 9.37 [12.76, 5.98]). High-speed running distance was greater in M1 (227m), than M5 (92m) (ES = 27.95 [37.68, 18.22]) and M10 (138m) (ES = 8.46 [11.55, 5.37]). Interestingly, the s-RPE response was higher in M1 (331au) in comparison to the last mesocycle (M10, 239au). HI showed minor variations across mesocycles and in days prior to the match. Every day prior to a match, all internal and external TL variables expressed significant lower values to other days prior to a match (p<0.01). In general, there were no differences between player positions.

Conclusions: Our results reveal that despite the existence of some significant differences between mesocycles, there were minor changes across the in-season period for the internal and external TL variables used. Furthermore, it was observed that MD-1 presented a reduction of external TL (regardless of mesocycle) while internal TL variables did not have the same record during in-season match-day-minus.

## Introduction

The knowledge of internal and external training load (TL) helps coaches to prevent increased levels of fatigue, and higher risk of illness and injury [1]. Also, it helps coaches to design an effective individual and group training periodization in elite team sports [2–7]. However, it is only recently that some studies have described the in-season training periodization practices of elite football teams in more detail, including a comparison of training days within weekly microcycles [4, 7–9]. As an example, Malone et al. [4] found that a lowering of TL in the last training day immediately before any given match differed from the other training days on several internal and external TL load variables such as session rated perceived exertion (s-RPE), plus total distance and average speed, respectively. The same authors stated that the need to win matches does not allow to reach of a specific peak for strength and conditioning [4]. In addition, some studies have shown limited variation through the in-season and have suggested that training in elite soccer has a regular load pattern [4, 5, 10, 11].

Moreover, several authors [1, 10, 12, 13] have claimed that it is also very important to monitor elite athletes’ health to provide further information concerning the details of player fatigue, stress, muscle soreness and sleep perception. These variables are commonly associated with psychophysiological stress responses, such as rating of perceived exertion or Hooper Index (HI) scores, also recognized as internal TL [13, 14]. On this issue, a valid and simple way to control internal TL is the session rating of perceived exertion (s-RPE) which showed correlations to the heart frequency training zones [15]. Furthermore, another way to quantity the level of fatigue, stress, s muscle soreness and the quality of sleep is the Hooper Index [12].

However, the simultaneous use of s-RPE and HI is limited. In fact, very few authors have studied the relationship between the use of the HI and s-RPE [10, 16]. Here, Clemente et al. [10] found a correlation between s-RPE and HI levels, and negative correlations between s-RPE and muscle soreness (p = −0.156), s-RPE and sleep (p = −0.109), s-RPE and fatigue (p = −0.225), ITL and stress (p = −0.188) and ITL and HI (p = −0.238) in 2-game weeks. On the other hand, Haddad et al. [16] failed to observe any association between HI and RPE. Therefore, further research is needed to clarify this issue, specifically to validate these results during in-season. Subsequently, it is also necessary to quantify the external TL that is associated with the total amount of workload performed during training sessions and/or matches [13–14]. According to Halson [17] and Casamichana et al. [18], one easy and practical way to control training response for each player (e.g. frequency, time, total distance and distances of different exercise training intensity) is time-motion analysis by using a global positioning system (GPS).

Nowadays, researchers study the data collected during short training microcycles of 1-2-3 weeks [9–10, 13, 19], in mesocycles consisting of 4–10 weeks [20–22] and during longer training periods of 3–4 months [18, 23] and 10-month periods [11]. However, most of these studies have provided limited information regarding the TL, using only the duration and RPE without the inclusion of other internal and external TL variables such as HI or data collected from GPS. In addition, few studies [4–5, 10] have attempted to quantify TL with respect to changes between mesocycles and microcycles (both overall and between player’s positions) across an in-season.

Finally, the literature is somewhat inconclusive about establishing differences in TL for player positions not only amongst training sessions but also during the in-season across a full competitive season regarding training sessions, but there is information related to match-play data that reveals some differences for player positions [4, 24]. Therefore, the purpose of this study was twofold: a) quantify external TL in an elite professional European soccer team that played UEFA competitions across ten months of the in-season 2015/16 and b) quantify the internal TL using s-RPE and HI. For this purpose, we divided the in-season into ten months, following Morgan et al. [11], and used the match day minus approach used by Malone et al. [4] for data analysis. Additionally, we also compared player positions for both situations. We hypothesized that training load is lower on training days closer to the next match and that the intensities and volume remain constant throughout the competitive period.

## Materials and methods

### Participants

Nineteen elite soccer players with a mean ± SD age, height and mass of 26.3 ± 4.3 years, 183.5 ± 6.6 cm and 78.5 ± 6.8 kg, respectively, participated in this study. The players belong to a team that participated in UEFA Champions League. The field positions of the players in the study consisted of four central defenders (CD), four wide defenders (WD), four central midfielders (CM), four wide midfielders (WM) and three strikers (ST). Inclusion criteria were regular participation in most of the training sessions (80% of weekly training sessions); the completion of at least 60 minutes in one match in the first half of the season and one match in the second half of the season. All participants were familiarised with the training protocols prior to the investigation and gave their written consent to be included in the project. The study was conducted according to the requirements of the Declaration of Helsinki and was approved by Ethics Committee of the Research Centre for Sports Sciences, Health and Human Development, Vila Real, Portugal.

### Design

TL data were collected over a 39-week period of competition where occurred 50 matches during the 2015–2016 annual season. The team used for data collection competed in four official competitions across the season, including UEFA Champion league, the national league and two more national cups from their own country. For the purposes of the present study, all the sessions carried out as the main team sessions were considered. This refers to training sessions in which both the starting and non-starting players trained together. Only data from training sessions were considered. Data from rehabilitation or additional training sessions of recuperation were excluded. This study did not influence or alter the training sessions in any way. Training data collection for this study was carried out at the soccer club’s outdoor training pitches. A total of 2981 individual training observations were collected during In-season. Total minutes of training sessions included warm-up, main phase and slow down phase plus stretching. A total of 349 individual observations contained missing data due to factors outside of the researcher’s control (eg, technical issues with equipment).

### Methodology

The in-season phase was divided into 10 mesocycles or 10 months, respectively, as used by Morgans et al. [11] and because the coaches and staff of the club work by months. Training data were also analysed in relation to the number of days away from the competitive one-match week (i.e., match day minus). In a week with only one match, the team typically trained five days a week (match day [MD] minus [–]; MD-5; MD-4; MD-3; MD-2; MD-1), plus one day after the match (MD+1). This approach was used by Malone et al. [4].

### External training load–training data

A portable global positioning system (GPS) units (Viper pod 2, STATSports, Belfast, UK) was used to monitor the physical activity of each player (external TL). This device provides position velocity and distance data at 10 Hz frequency. The use of the device by each player is reported in Oliveira et al. [25]. All players wore the same GPS device for each training session in order to avoid inter unit error [26]. Previously, this GPS system have been able to provide valid and reliable estimates of instantaneous and constant velocity movements during linear, multidirectional and soccer-specific activities [27] Following recommendations by Maddison & Ni Mhurchu [28], all devices were activated 30 minutes before data collection to allow the acquisition of satellite signals and synchronise the GPS clock with the satellite’s atomic clock. GPS data were then downloaded using the respective software package (Viper PSA software, STATSports, Belfast, UK) and were clipped to involve the main team session (i.e. the beginning of the warm up to the end of the last organised drill). The number of satellites visualized by this unit, as well as the horizontal dilution of position, is not reported by this GPS model, and therefore, are not reported in this study.

The metrics selected for the study were total duration of training session, total distance, high-speed distance (HSD, above 19Km/h) and average speed (AvS).

### Internal training load–training data

Approximately 30 min before each training session, each player was asked to provide the Hooper Index scores. This index includes four categories: fatigue, stress, muscle soreness and quality of sleep of the night that preceded the evaluation. It was used the Hooper index scale of 1–7, in which 1 is very, very low and 7 is very, very high (for stress, fatigue and muscle soreness levels) and 1 is very, very bad and 7 is very, very good (for sleep quality). The summation of the four subjective ratings is the Hooper Index [12].

Thirty minutes following the end of each training session, players were asked to provide an RPE rating, 0–10 scale [29]. Players were prompted for their RPE individually using a custom-designed application on a portable computer tablet. The player selected their RPE rating by touching the respective score on the tablet, which was then automatically saved under the player’s profile. This method helped minimise factors that may influence a player’s RPE rating, such as peer pressure and replicating other player’s ratings [30]. Each individual RPE value was multiplied by the session duration to generate a session-RPE (s-RPE) value [21, 31, 32]. Further details regarding s-RPE are reported in Oliveira et al. [25].

### Statistical analysis

Data were analysed using SPSS version 22.0 (SPSS Inc., Chicago, IL) for Windows statistical software package. Initially, descriptive statistics were used to describe and characterize the sample. Shapiro-Wilk and the Levene tests were used to assumption normality and homoscedasticity, respectively. ANOVA was used with repeated measures with Bonferroni post hoc, once variables obtained normal distribution (Shapiro-Wilk>0.05), to compare 10 mesocycles and to compare days away from the competitive match fixture. Also, it was used ANOVA Friedman and Mann-Whitney tests were used for the variables that not obtained normal distribution to compare different moments and different player positions. Results were significant with p≤0.05. The effect-size (ES) statistic was calculated to determine the magnitude of effects by standardizing the coefficients according to the appropriate between-subjects standard deviation and was assessed using the following criteria: <0.2 = trivial, 0.2 to 0.6 = small effect, 0.6 to 1.2 = moderate effect, 1.2 to 2.0 = large effect and >2.0 = very large [33]. The associations between s-RPE and HI scores were tested with Spearman correlation. Data are represented as mean ± SD.

## Results

### In-season mesocycle analysis

**Fig 1:**
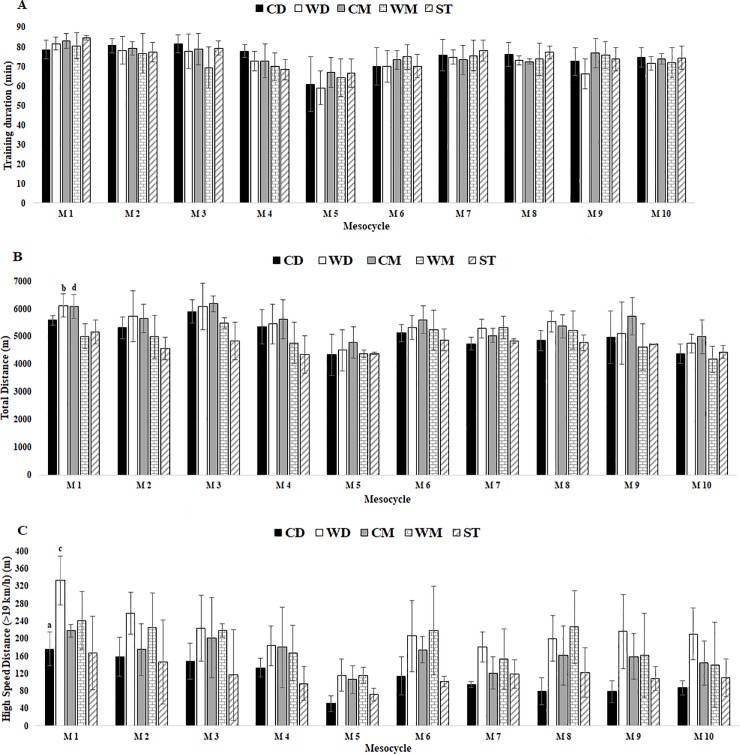
External TL data for training duration, total distance and HSD in respect to mesocycles between player positions. Abbreviations: (A) training duration; (B) total distance; (C) HSD; (CD), central defenders; (WD), wide defenders; (CM), central midfielders; (WM), wide midfielders; (ST), strikers. a denotes significant difference in CD versus WD, (b) denotes significant difference in WD versus WM, (c) denotes significant difference in WD versus ST, (d) denotes significant difference CM versus WM, all P < 0.05.

The results indicate that duration of training sessions (Table 1) had more minutes in M1 than in other mesocycles and M5 was the lowest. There were no differences between player positions during in-season (Fig 1).

**Table 1:** External Training Load Data during the ten mesocycles for squad average, Mean ± SD.

Mesocycle (M)	Number of matches	Number of training sessions	Training Duration (min)	Total Distance (m)	Average speed (m/min)	HSD (m)
M1	4	16	81.6±1.1 ^c^^,^ ^d^^,^ ^e^^,^ ^g^^,^ ^h^^,^ ^i^^,^ *	5589.1±100.1 ^d^^,^ ^i^^,^ *	68.6±1.1	227.0±13.7 ^d^^,^ ^e^^,^ ^f^^,^ ^g^^,^ ^h^^,^ ^i^^,^ *
M2	5	20	78.4±1.6 ^d^^,^ ^i^^,^ *	5248.2±156.2 ^b^^,^ ^i^^,^ *	66.8±0.9 ^b^^,^ *	192.3±17.0 ^d^^,^ ^g^^,^ *
M3	4	18	77.4±1.9 ^d^	5691.4±132.1 ^d^^,^ ^f^^,^ ^i^^,^ *	74.0±1.7 ^i^^,^ *	181.9±18.9 ^d^^,^ *
M4	5	18	72.3±1.6	5111.4±173.9	70.7±2.2	152.2±15.4 ^d^^,^ *
M5	6	20	63.6±2.4 ^f^^,^ ^g^^,^ ^i^^,^ *	4473.5±136.4 ^e^^,^ ^f^^,^ *	71.0±2.1 ^i^^,^ *	92.3±6.6 ^e^^,^ ^f^^,^ ^g^^,^ *
M6	8	20	71.7±1.8	5231.8±123.0 ^i^^,^ *	73.2±1.7 ^i^^,^ *	162.9±15.3
M7	5	19	75.5±1.7	5041.9±70.5 ^i^^,^ *	67.2±1.9	133.6±10.3
M8	4	20	74.5±1.2	5149.5±112.5 ^i^^,^ *	69.3±1.3 ^i^^,^ *	157.8±15.4
M9	7	18	72.9±1.8	5026.7±204.1	69.0±2.1 ^i^^,^ *	144.8±15.9
M10	4	20	73.3±1.3	4545.4±111.7	62.2±1.6	138.5±14.7

min = minutes; m = meters; HSD = high-speed distance.

^a^ denotes difference from M2

^b^ denotes difference from M3

^c^ denotes difference from M4

^d^ denotes difference from M5

^e^ denotes difference from M6

^f^ denotes difference from M7

^g^ denotes difference from M8

^h^ denotes difference from M9

^i^ denotes difference from M10

all P < 0.05

* very large effect.

For external load, total distance tended to decrease during in-season. M1 and M3 obtained a greater distance. There were significant differences between player positions in M1 for WD vs WM (ES = 4.87 [2.92, 6.82]), CM vs WM (ES = 5.07 [3.06, 7.09) (Fig 1).

Regarding average speed, M3 reached the highest value and M10 reached the lowest.

High-speed distance reached the highest value in M1 and lowest in M5. There were significant differences between player positions in M1 for CD vs WD (ES = 5.01 [3.02, 7.00]).

**Fig 2:**
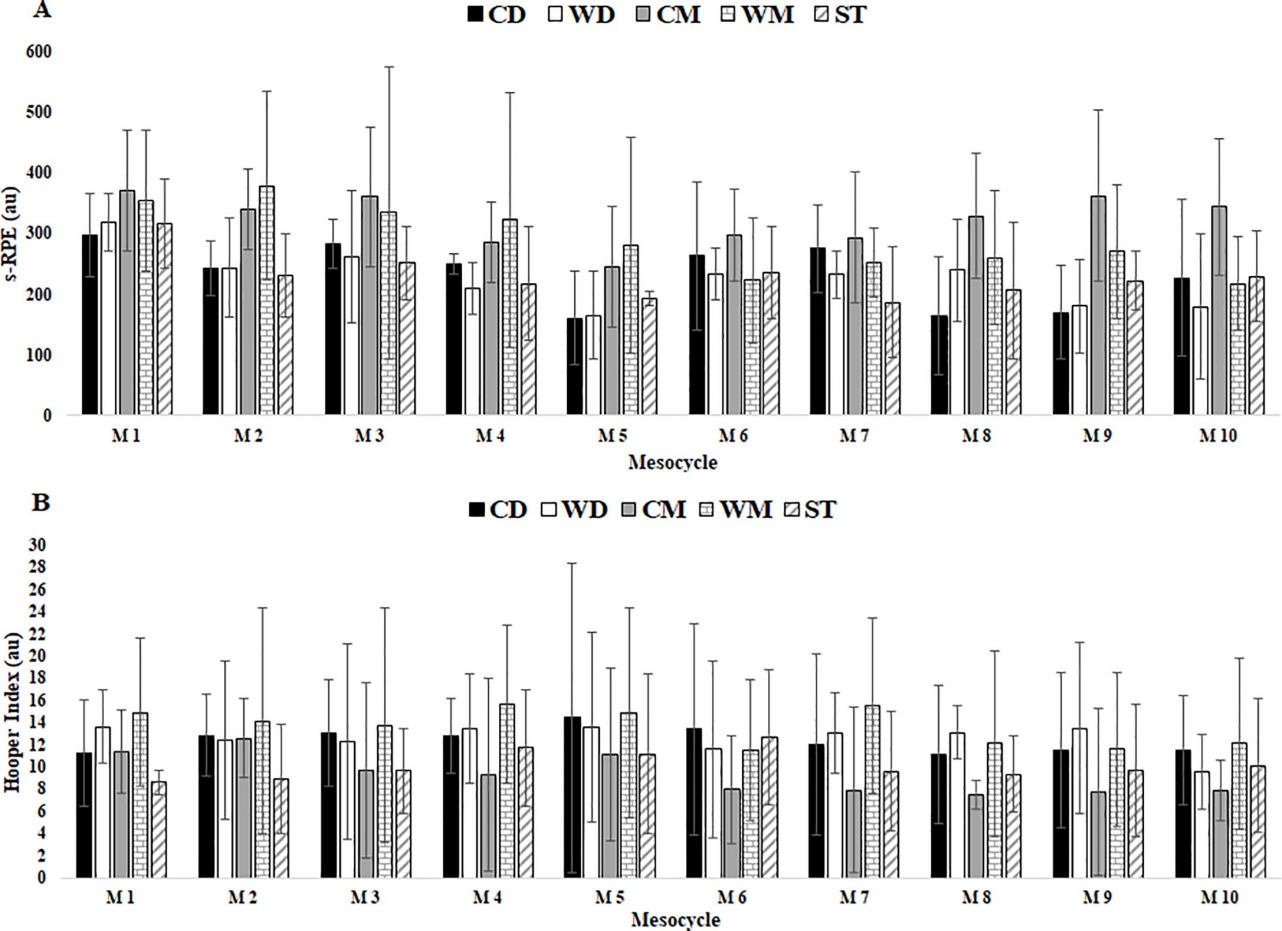
Internal TL data s-RPE and HI in respect to mesocycles between player positions. Abbreviations: (A) s-RPE; (B) HI; (CD), central defenders; (WD), wide defenders; (CM), central midfielders; (WM), wide midfielders; (ST), strikers. a denotes significant difference in CD versus WD, (b) denotes significant difference in WD versus WM, (c) denotes significant difference in WD versus ST, (d) denotes significant difference CM versus WM, all P < 0.05.

For internal load (Table 2), s-RPE was higher in M1 with a tendency to decrease until the end of the season -, M10. There were no differences between player positions during in-season (Fig 2).

**Table 2:** Internal Training Load Data during the ten mesocycles for squad average, Mean ± SD.

Mesocycle	s-RPE (au)	Fatigue (au)	Stress (au)	Muscle Soreness (au)	Sleep quality (au)	HI (au)
M1	331.9±21.6 ^a^^,^ ^b^^,^ ^c^	3.0±0.3	3.2±0.2	3.0±0.3	2.8±0.2	11.9±0.8
M2	287.3±22.6 ^a^	3.2±0.3	3.2±0.2	3.2±0.2	2.7±0.2	12.1±0.8
M3	298.4±33.2	3.1±0.3	3.0±0.1	3.1±0.3	2.5±0.2	11.7±0.7
M4	256.9±26.6	3.4±0.2	3.0±0.2	3.3±0.2	2.9±0.3	12.6±0.7
M5	208.6±25.9	3.6±0.2	3.2±0.2^c^^,^ *	3.4±0.3	2.8±0.3	13.0±0.7 ^c^^,^ *
M6	250.5±22.1	3.3±0.3	2.8±0.2	3.1±0.3	2.2±0.2	11.4±0.9
M7	247.8±20.4	3.2±0.4	3.0±0.3	3.1±0.4	2.4±0.2	11.6±1.1
M8	239.8±25.8	2.9±0.3	2.5±0.2	2.8±0.3	2.4±0.3	10.6±0.8
M9	240.8±25.5	3.0±0.3	2.7±0.2	2.8±0.4	2.3±0.2	10.8±0.8
M10	239.3±26.7	3.0±0.4	2.4±0.3	2.6±0.3	2.3±0.3	10.2±0.9

M = mesocycle (1, 2, 3, etc.); s-RPE = session rating of perceived effort; HI = Hooper index; au = arbitrary units.

^a^ denotes difference from M5

^b^ denotes difference from M8

^c^ denotes difference from M10, all P < 0.05

* very large effect.

HI had fewer variations during the in-season, reaching the highest value in M5 and the lowest value in M10. Also, Stress category revealed the same results between M5 and M10. There were no significant differences between player positions for HI scores (Fig 2).

There were associations between HI scores and s-RPE, HI scores and external TL variables, and S-RPE and external TL variables, but few correlations were found: stress and total distance in M2 (-0.634, p<0.01); fatigue and s-RPE in M9 (0.589, p<0.05); muscle soreness and s-RPE in M9 (0.487, p<0.05); fatigue and s-RPE in M11 (0.469, p<0.05); and HI total score and total distance in M11 (0.489, p<0.05).

### In-season match-day-minus training comparison

**Fig 3:**
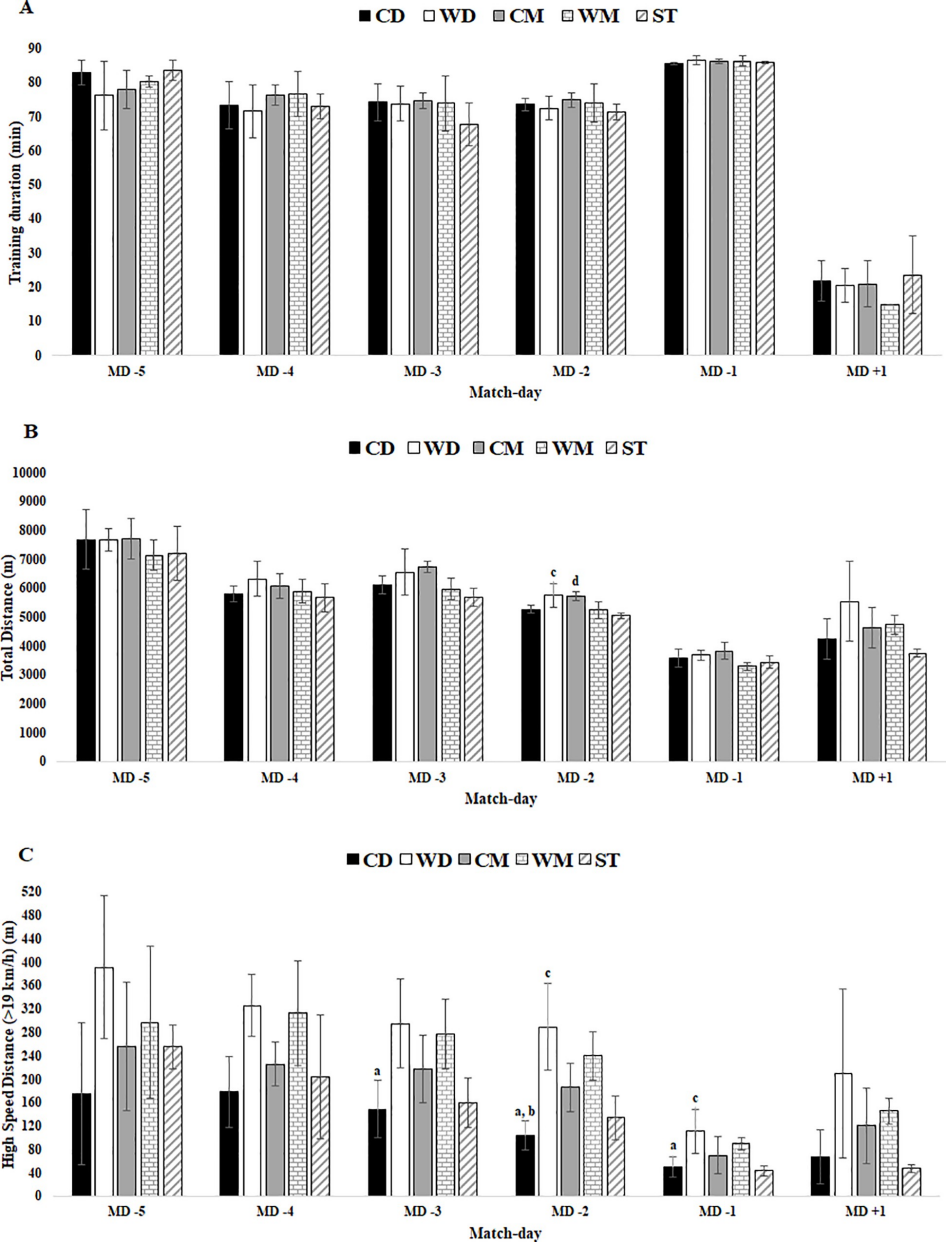
External TL data for training duration, total distance and HSD in respect to days before a competitive match between player positions. Abbreviations: A) training duration; (B) total distance; (C) HSD; (CD), central defenders; (WD), wide defenders; (CM), central midfielders; (WM), wide midfielders; (ST), strikers. (a) denotes significant difference in CD versus WD, (b) denotes.

The duration of training sessions (Table 3) in MD-1 and MD-5 was the second highest was the highest. MD+1 presented the lowest training duration. No differences were found between players positions (Fig 3).

**Table 3:** External Training Load Data during the MD minus for squad average, Mean ± SD.

MD	Duration (min)	Total Distance (m)	Average speed (m/min)	HSD (m)
MD-5 (n = 24)	80.2±1.3 ^b^^,^ ^c^^,^ ^d^^,^ ^e^^,^ *	7482.0±173.1 ^a^^,^ ^b^^,^ ^c^^,^ ^d^^,^ ^e^^,^ *	94.1±3.0 ^a^^,^ ^c^^,^ ^d^^,^ ^e^^,^ *	274.8±26.0 ^c^^,^ ^d^^,^ ^e^^,^ *
MD-4 (n = 20)	74.2±1.4 ^d^^,^ ^e^^,^ *	5943.9±105.4 ^c^^,^ ^d^^,^ ^e^^,^ *	80.4±1.2 ^c^^,^ ^d^^,^ ^e^^,^ *	249.3±16.3 ^c^^,^ ^d^^,^ ^e^^,^ *
MD-3 (n = 24)	72.8±1.3 ^d^^,^ ^e^^,^ *	6205.6±106.4 ^c^^,^ ^d^^,^ ^e^^,^ *	85.3±1.3 ^c^^,^ ^d^^,^ ^e^^,^ *	219.7±13.7 ^c^^,^ ^d^^,^ ^e^^,^ *
MD-2 (n = 24)	73.2±0.8 ^d^^,^ ^e^^,^ *	5404.7±59.2 ^d^^,^ ^e^^,^ *	73.9±0.8 ^d^^,^ ^e^^,^ *	190.4±11.1 ^d^^,^ ^e^^,^ *
MD-1 (n = 24)	86.1±0.2 ^e^^,^ *	3564.7±55.6 ^e^^,^ *	41.4±0.6 ^e^^,^ *	72.4±5.7 ^e^^,^ *
MD+1 (n = 20)	20.4±1.5	4576.7±184.8	243.8±16.4	117.8±17.8

MD- = matchday minus (5. 4. 3. 2. 1); MD+1 = matchday plus 1; min = minutes; m = meters; HSD = high-speed distance.

^a^ denotes difference from MD-4.

^b^ denotes difference from MD-3.

^c^ denotes difference from MD-2.

^d^ denotes difference from MD-1.

^e^ denotes difference from MD+1.

all P < 0.01

* very large effect.

For external load, total distance reached the highest value in MD-5 and the lowest in MD-1. Regarding player positions (Fig 3), there were significant differences in MD-2 between WD vs ST (5.13 [9.19, 1.07]) and CM vs ST (5.01 [9.01, 1.02]).

Average speed reached the highest value in MD-5 and the lowest in MD-1. No differences were found between player positions (Fig 2).

High-speed distance reached the highest value in MD-5 and the lowest in MD-1. In MD-3 there were significant differences between player positions (Fig 2) for CB vs WD (4.94 [1.01, 8.89]). In MD-2 there were significant differences between CD vs WD (7.81 [2.05, 13.57]), CD vs WM (5.74 [1.31, 10.17]) and WD vs ST (6.02 [10.62, 1.41]). In MD-1 there were significant differences between CD vs WD (4.93 [0.99, 8.86]) and WD vs ST (5.03 [1.03, 9.04]).

**Fig 4:**
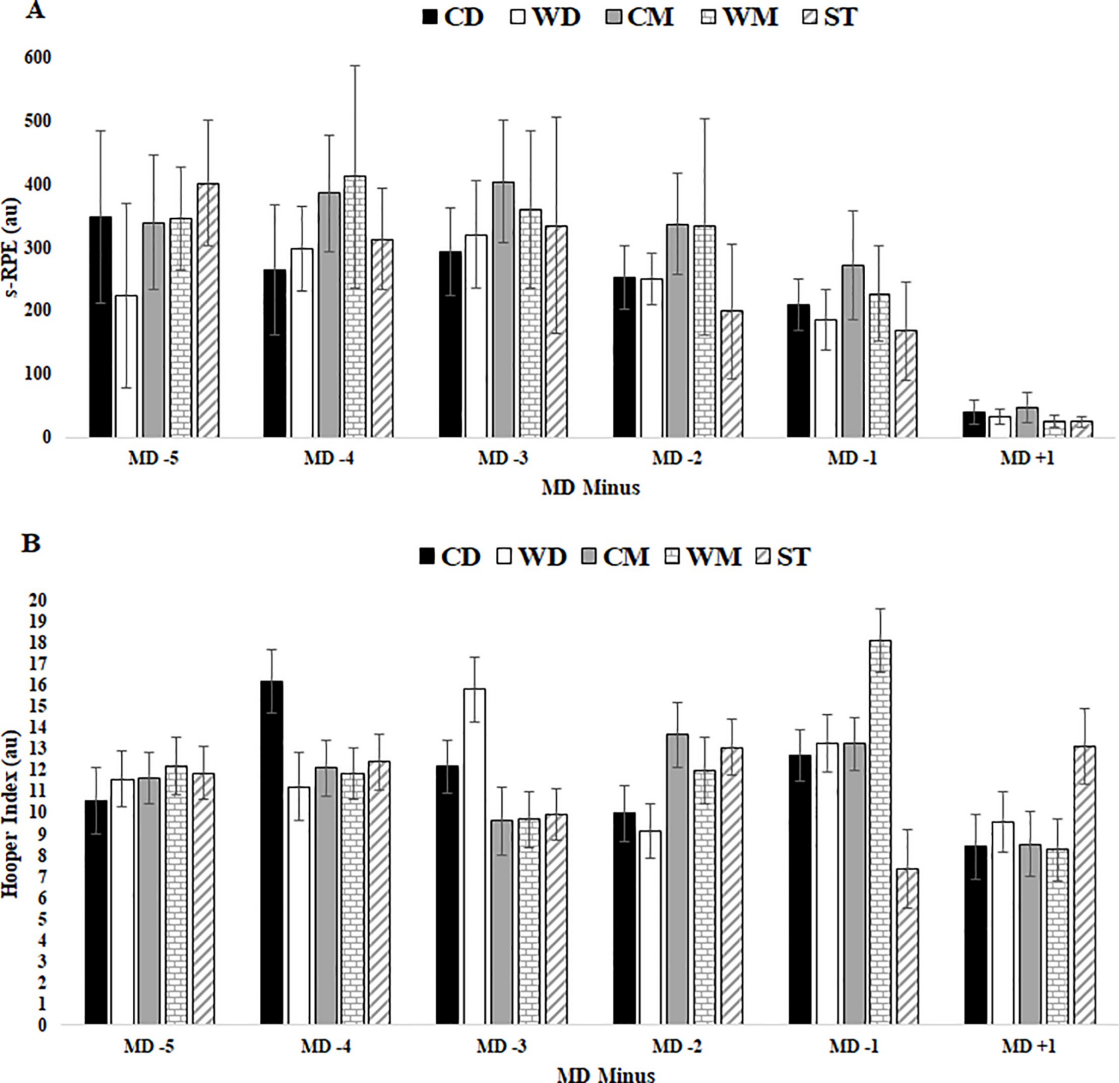
Internal TL data for s-RPE and HI in respect to days before a competitive match between player positions. Abbreviations: A) s-RPE; (B) HI; (CD), central defenders; (WD), wide defenders; (CM), central midfielders; (WM), wide midfielders; (ST), strikers. (a) denotes significant difference in CD versus WD, (b) denotes.

For internal load (Table 4), s-RPE reached the highest value in MD-3 and revealed a tendency to decrease until MD-1. The lowest were found in MD+1. No differences were found between player position (Fig 4).

**Table 4:** Internal Training Load Data during the MD minus for squad average, Mean ± SD.

MD	s-RPE (au)	Fatigue (au)	Stress (au)	Muscle Soreness (au)	Sleep quality (au)	HI (au)
MD-5	331.7±27.0 ^d^^.^ ^e^^,^ **	3.1±0.8^e^^,^ **	2.3±0.2^e^^,^ **	2.9±0.6^e^^,^ **	1.9±0.9^a^^,^^b^^,^^c^^,^^d^^,^^e^	10.2±0.7 ^e^^,^ **
MD-4	334.4±25.8 ^c^^.^ ^d^^.^ ^e^^,^ **	2.9±0.6^e^^,^ **	2.9±0.7^e^^,^ *	2.8±0.6^e^^,^ **	2.6±0.5 **	11.1±0.6 ^e^^,^ **
MD-3	342.4±25.3 ^d^^.^ ^e^^,^ **	2.8±0.6^e^^,^ **	2.9±0.6^e^^,^ **	2.9±0.5^e^^,^ **	2.5±0.6 *	11.1±0.6 ^e^^,^ **
MD-2	274.3±23.2 ^d^^.^ ^e^^,^ **	3.0±0.5^e^^,^ **	2.7±0.6^e^^,^ **	3.0±0.6^e^^,^ **	2.6±0.7 *	11.3±0.6 ^e^^,^ **
MD-1	212.3±15.5 ^e^^,^ **	2.9±0.6^e^^,^ **	2.6±0.6^e^^,^ **	2.9±0.6^e^^,^ **	2.5±0.6 *	10.9±0.6 ^e^^,^ **
MD+1	33.6±3.7	4.4±0.7	3.9±0.2	4.4±0.7	2.7±0.9 *	15.4±0.7

MD- = matchday minus (5. 4. 3. 2. 1); MD+1 = matchday plus 1; s-RPE = session rating of perceived effort; HI = Hooper index; au = arbitrary units.

^a^ denotes difference from MD-4.

^b^ denotes difference from MD-3.

^c^ denotes difference from MD-2.

^d^ denotes difference from MD-1.

^e^ denotes difference from MD+1.

all P < 0.01

* large effect

** very large effect.

HI and all categories had few variations during the MD minus with the exception of MD+1 where the highest values were found. No differences were found between player positions (Fig 4).

## Discussion

The purpose of the present study was to quantify the internal and external TL carried out by an elite soccer team during the in-season (10 mesocycles). The main findings of the study are related to similar training load during in-season, but HSD and s-RPE were higher in the first mesocycle. Also, external TL until MD-1 while internal TL variables did not present the same pattern. In addition, HI remained constant for all mesocycle and training sessions with the exception for the following day of the match.

### In-season mesocycle analysis

For external TL variables, it was observed that the players covered a greater total distance at the start (M1 and M3) compared to the final mesocycle (M10) of the in-season, with an estimated difference of 1044m and 1146m, respectively. The higher distances covered at the beginning of the in-season may be due to the coaches still having some emphasis on physical conditioning immediately after the pre-season. In addition, the lower values in distance covered for M10 could be associated with the in-season ending and consequently a reduction in external TL.

According to Impellizzeri et al. [21] and Alexiou & Coutts [23], the competitive matches represent the greatest TL that soccer players typically experience. In addition, Malone et al. [4] and Los Arcos et al. [34] reported that total distance values were significantly higher at the start of the annual in-season compared to the final stage 1304 (434–2174) m, ES = 0.84 (0.28–1.39) and (ES = from– 0.56 to -1.20), respectively. These previous data corroborate our results because it was possible to observe higher values in M1 compared to M10, although M5 had the lowest values for total distance (Table 1).

The present data suggest that in-season variability in TL is very limited and only minor decrements in TL across the in-season might occur. Apparently, this TL maintenance during the in-season could be associated with the importance of the recovery activities after the matches and the decisions made to reduce TL until the next match [35]. Furthermore, elite European soccer teams training programmes remain constant during all mesocycles of the in-season and corroborate the suggestion made by Malone et al. [4] because there is a need to win matches that does not allow the reaching of a specific peak for strength and conditioning.

The average total distance covered was 5111m (4473-5691m) which was similar to the 5181m value reported by Malone et al. [4] and slightly higher than those reported by Gaudino et al. [20] (3618-4133m). However, both the distances covered in the present study and in Gaudino et al. [20] study fell short in comparison to those reported by Owen et al. [19] (6871m) because their study only included data from training sessions. This means that the study conducted by Owen et al. [19] reported higher distances covered even with lower training sessions. In terms of high-speed distance, the values (average 118m) fall within the range of that of Gaudino et al. [20] (88–137m) across different positions.

The results indicate that TL variables demonstrated limited relevant variation between player positions (see Figs 1 and 2). It seems that competitive matches have been quantified as the most demanding session (i.e. greatest TL) of the week [7, 24, 25, 34, 36]. Previous work corroborated this statement, although player position was not analysed [25]. For instance, Di Salvo et al. [37] reported that CM generally cover more distances compared to other positions during competitive matches. This result corroborates the current results because CM (5502m) covered more total distance than CD (5052m), WD (5388m), WM (4918m) or ST (4694m), but without statistical significance. In addition, when we compared the distance covered in high-speed running zones (zones 4+5) during in-season mesocycle analysis to positions played, a significant difference was found between positions only for M1 when comparing CD vs WD and WD vs WM. There was no other difference between player positions in all mesocycles (Fig 1). These results suggest that the WD (212.7m) and WM (186,8m) positions resulted in higher effort (>19km/h) during training when compared to all other positions (CD = 112.2, CM = 164.1, ST = 116.1m). Further, every position saw similar efforts at low speed distance (CD = 4563.7; WD = 4724.5, CM = 4767.8, WM = 4340.4, ST = 4233.3m) which is in opposition to other studies [24, 37, 38].

Regarding internal TL, the s-RPE response was higher in M1 (331au) in comparison to the last mesocycle (M10, 239au) which is in line with data from external TL total distance and HSD variables. However, it is relevant to consider that this also was the mesocycle with higher training duration. Furthermore, it was found that in the middle of the season (M5) there was a lower response (208au) for this parameter. This finding could be associated with some interruption for TL carried out during training sessions due to the Christmas period and with an increase in the number of matches played in M5 (6 matches). In general, there were no differences between player positions (see Fig 1). Therefore, it appears that there is no marked variation in internal TL across 10 mesocycles during the in-season. Some studies [4, 5, 10, 11] have also reported the limited relevant variation in TL across the in-season. This seems to suggest that professional soccer daily training practices follow a regular load pattern because they are linked to higher congestive periods of matches. Furthermore, the importance of the recovery activities following matches and the decisions made to reduce TL between matches to prevent fatigue during this period can also play an important role in this constant TL [35].

Moreover, the data provides relevant information to quantify internal TL, measured by s-RPE during microcycles and mesocycles. This may provide relevant information to establish guidelines for soccer training periodization. The average of s-RPE during microcycles TL was 254.8au (range 33-342au). These values are lower than those reported by Scott et al. [22] (297au: range 38-936au), but similar to Jeong et al. [39] study: 174-365au. for elite Korean soccer players. The s-RPE values were also lower than the 462au of semi-professional soccer players reported by Casamichana & Castellano [18]. Another explanation for the lower values could be related to the number of matches during each week and amongst mesocycles. It should be reemphasised that we studied a top-class elite professional European soccer team. The range of s-RPE for mesocycles of the in-season was 208-331au. Overall it would appear that in comparison to top elite soccer players, the internal TL employed by our study falls within the boundaries of what has been previously observed [18, 22, 39].

Haddad et al. [16] suggested that s-RPE is not sensitive to the subjective perception of fatigue, muscle soreness or stress levels [16]. In contrast, however, Clemente et al. [10] stated that s-RPE could be a reliable tool to quantify the internal TL and therefore could be a good indicator for coaches and for practical applications in team sports training. Data presented in the current experiment seems to corroborate this statement, indicating that s-RPE can be an effective tool to measure the intensity and duration of training session in elite European soccer teams. On this subject, some studies have stated that RPE may be a physiological and volatile construct that could be different according to the cognitive focus of the player [40–42]. Nevertheless, Renfree et al. [43] reported that RPE can be dissociated from the physiological process through a variety of psychological mechanisms. Therefore, RPE could be an oversimplification of the psychophysiological perceived exertion and a non-conclusive measure for capturing a wide range of sensations experience [40, 41, 43]. Another major point is that RPE was collected 30 min after the end of each training session and it would be pertinent to check if there is some variation during the training session, as contended by Ferraz et al. [41]. These arguments may justify the fact that there were no differences in s-RPE between training days as well as the absence of a relationship with the external TL results.

HI remained similar during 10 mesocycles. In addition, comparing player positions, there were no differences for HI scores; this was not supported by Clemente et al. [10] although their study was based on data from one vs two-matches week (p< 0.05). To the best of our knowledge, this is the first study to analyse HI scores during an entire in-season. Clemente et al. [10] showed that central defenders (12.46 ± 2.54) and wide midfielder (12.42 ± 3.44) had higher values of HI scores than strikers (12.18 ± 4.84) and wide defenders (12.16 ± 3.04). Centre midfielders had the lowest HI scores (10.34 ± 3.87). Despite these, the authors found several significant differences between positions but, in general, these values were small. A possible explanation for these non-consensual results could be associated with the differences in soccer TL.

In soccer training, due to the extensive use of small-sided matches and the different physical (e.g. running) requirements associated with each position [37, 44, 45], training demands can be markedly different between individuals [13, 46, 47]. This hypothetical difference in TL could be amplified considering that only 11 players can start each official match, and therefore a considerable number of players per team are not exposed to the TL of the match.

As suggested by Clemente et al. [10] study, we also correlated HI scores with s-RPE and external TL variables, and some correlations could be observed: stress and total distance in M2 (-6.34, p<0.01); fatigue and s-RPE in M9 (0.589, p<0.05); muscle soreness and s-RPE in M9 (0.487, p<0.05); fatigue and s-RPE in M11 (0.469, p<0.05); and HI total score and total distance in M11 (0.489, p<0.05). These results are not in line with the literature, which suggests non-significant correlations (r = 0.20) between s-RPE and perceived quality of sleep (from the Hooper questionnaire) [10, 48]. However, Thorpe et al. [49] reported associations between s-RPE and perceived fatigue, but not with perceived quality of sleep. It is important to note that this last study analysed data for short periods of training (microcycles). Therefore, since our study also comprised longer periods of training, we can assume that this could have influenced the current results.

### In-season match-day-minus training comparison

In the present study, we also investigated the TL pattern in respect to number of days prior to a one- match week during the in-season phase.

For external TL, our data provided the following pattern by decreasing values from until MD-1: MD-5 > MD-4 < MD-3 > MD-2 > MD-1 for total distance and average speed, MD-5 > MD-4 > MD-3 > MD-2 > MD-1 for HSD (Table 2). Our results are not in line with elite English Premier League players for total distance and average speed, where it was found a lowering of the load only in MD-1 [4].

We also observed a noticeable consistent variation in external TL, total distance covered, in MD-1 when the load was significantly reduced in comparison with the rest of the training days. Our data corroborates with some studies [4, 8, 49].

Finally, MD+1 revealed significant result despite the limited training duration (~20 min). The average speed and HSD has higher values than all other match days minus. One argument that can justify these results could be the high-intensity applied by the coach (which was not controlled in this study). Another explanation is related to the context, competitive schedule and the objectives defined for TL management, once MD+1 had little duration (20min). Another possible justification could be associated with a training session of recuperation with lower load for starters and a “normal” training session for non-starters.

When we compared HSD (above 19Km/h) during in-season match-day-minus by positions, a significant difference was found between positions when comparing WD vs ST and CD vs WD, CD vs WM in MD-2 in MD-2. In addition, when we compared total distance covered, a significant difference could be observed between CD (149m) vs WD (295m) in MD-3, CD (103m) vs WD (289m) in MD-2 and CD (49m) vs WD (111m) in MD-1; CD (103m) vs WM (240m), WD (289m) vs ST (134m) in MD-2; and also WD (111m) vs ST (43m) in MD-1 (Fig 2). These results are in line with other studies [24, 37–38] that reported that CM players have consistently been found to cover more distance in general while WM players cover more distances at high-intensity running speed.

Regarding match days, Reilly & Thomas [50] and Rienzi et al. [51] stated that higher distances are covered by midfield players (11.5km); however, Bangsbo [52] reported that elite defenders and strikers covered approximately the same distance (10–10.5km). This may be due to the nature and role of the position inside the team, as well as coaching strategy and/or game plan. During training sessions, the coach or the conditioning staff may find it advantageous to model training to elicit similar effort or experience the same training load regardless of position.

For internal TL, s-RPE data presented a non-perfect pattern by decreasing values from until MD-1: MD-5 < MD-4 < MD-3 > MD-2 > MD-1 for s-RPE (Table 2), but none between player positions (Fig 2). We also observed a noticeable consistent variation in s-RPE on MD-1 in elite soccer players, when the load was significantly reduced in comparison with the rest of the training days [4, 8, 49]. In addition, the data presented by s-RPE is associated with external TL variation.

Furthermore, HI scores revealed no variation in days prior to the match. These results are in line with those reported by Haddad et al. [16], where it was suggested that fatigue, stress, muscle soreness and sleep are not major contributors of perceived exertion during traditional soccer training without excessive TL. Our results also do not support Hooper and Mackinnon [12] study because self-reported ranking of well-being does not allow the provision of efficient mean of monitoring internal TL. In fact, the only exception was sleep quality category which revealed the lowest value and therefore bad sleep quality in MD-5. This higher value could be associated to the stimulus imposed by the previous match. It is relevant to remember that microcycles had different week-patterns and consequently, MD-5 could also be related to the following day of the match.

In opposition to the results presented for external in MD+1, internal TL, s-RPE has a lower value than all other match days (33.6 au) but HI has a higher value than all other match days (15au) (Table 1). These results are associated with an accumulative high-intensity training session between MD-5 and MD-2 and also supports the claim that matches represent the most demanding workload of each week [7, 24, 25, 34, 36].

### Practical applications and limitations

This study provides useful information relating to the TL employed by an elite European soccer team that played in a European Competition. It provides further evidence of the value of using the combination of different measures of TL to fully evaluate the patterns observed across the in-season. For coaches and practitioners, the study generates reference values for elite players which can be considered when planning training sessions. However, it is important to remember that the in-season match-day-minus training comparison was analysed by mean values and microcycles/weeks (7-day period) of the in-season have different patterns, as mentioned before. Another limitation is related to the numerous true data points missing across the 39-week data collection period due to several external factors beyond our control (e.g. technical issues with equipment, player injuries, and player transfers). Finally, GPS technology used in this study does not allow to report the horizontal dilution of precision and for that reason the findings regarding external TL need to be interpreted considering such a limitation as stated in Beato et al. [27].

## Conclusions

In summary, we provide the first report across 10 mesocycles of an in-season that included HI scores and s-RPE to measure internal TL plus distances covered at different intensities measured by GPS, in elite soccer players that played European competitions. Our results reveal that although there are some significant differences between mesocycles, there was minor variation across the season for the internal and external TL variables used. In addition, it was observed that MD-1 presented a reduction of external TL during in-season match-day-minus training comparison (regardless of mesocycle) (i.e. reduction of total distance, HSD and AvS) and internal TL (s-RPE). However, the internal TL variable, HI did not change, except for MD+1. This study also provided ranges of values for different external and internal variables that can be used for other elite teams.
