# Correction: In-season internal and external training load quantification of an elite European soccer team

**DOI:** 10.1371/journal.pone.0303763

**Published:** 2024-05-09

**Authors:** Rafael Oliveira, João P. Brito, Alexandre Martins, Bruno Mendes, Daniel A. Marinho, Ricardo Ferraz, Mário C. Marques

In the fifth paragraph of In-season mesocycle analysis subsection of Discussion, there is an error in the sixth sentence. The correct sentence is: In addition, when we compared the distance covered in high-speed high-speed distance (>19 km/h) during in-season mesocycle analysis to positions played, a significant difference was found between positions only for M1 when comparing CD vs WD and WD vs WM.

In the last paragraph of In-season mesocycle analysis subsection of Discussion, there is an error in the first sentence. The correct sentence is: As suggested by Clemente et al. (2017) study, we also correlated HI scores with s-RPE and external TL variables, and some correlations could be observed: stress and total distance in M1 (-6.34, p < 0.01); fatigue and s-RPE in M8 (0.589, p < 0.05); muscle soreness and s-RPE in M8 (0.487, p < 0.05); fatigue and s-RPE in M10 (0.469, p < 0.05); and HI total score and total distance in M10 (0.489, p < 0.05).

In Fig 3, the legend is incomplete. Please see the correct and complete legend of Fig 3 here.

**Fig 3:**
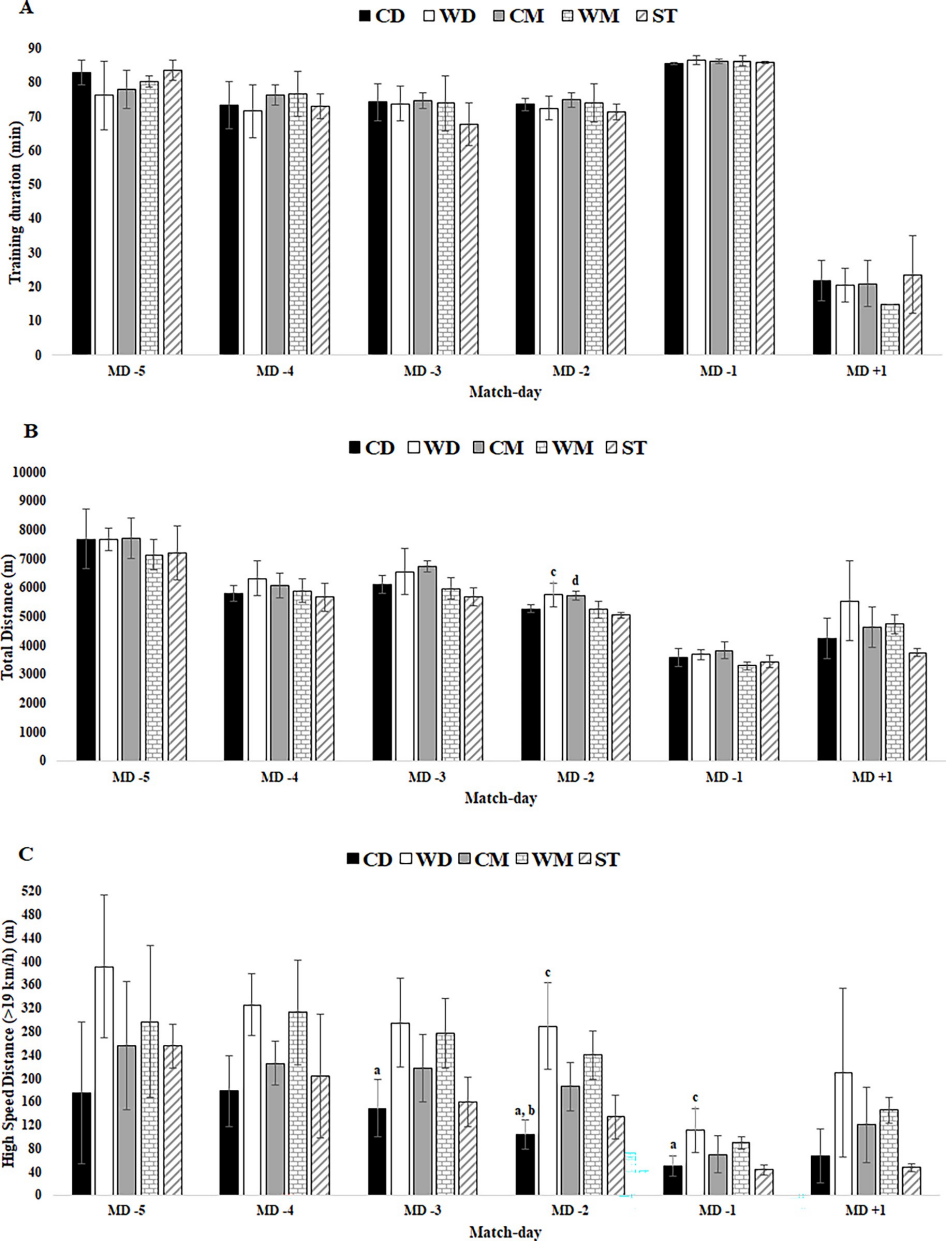
External TL data for training duration, total distance and HSD in respect to days before a competitive match between player positions. Abbreviations: A) training duration; (B) total distance; (C) HSD; (CD), central defenders; (WD), wide defenders; (CM), central midfielders; (WM), wide midfielders; (ST), strikers. (a) denotes significant difference in CD versus WD, (b) denotes significant difference in CD versus WM; (c) denotes significant difference in WD versus ST; (d) denotes significant difference in CM versus ST.

In Fig 4, the image for Hopper Index did not match with the results. Please see the correct Fig 4 here.

**Fig 4:**
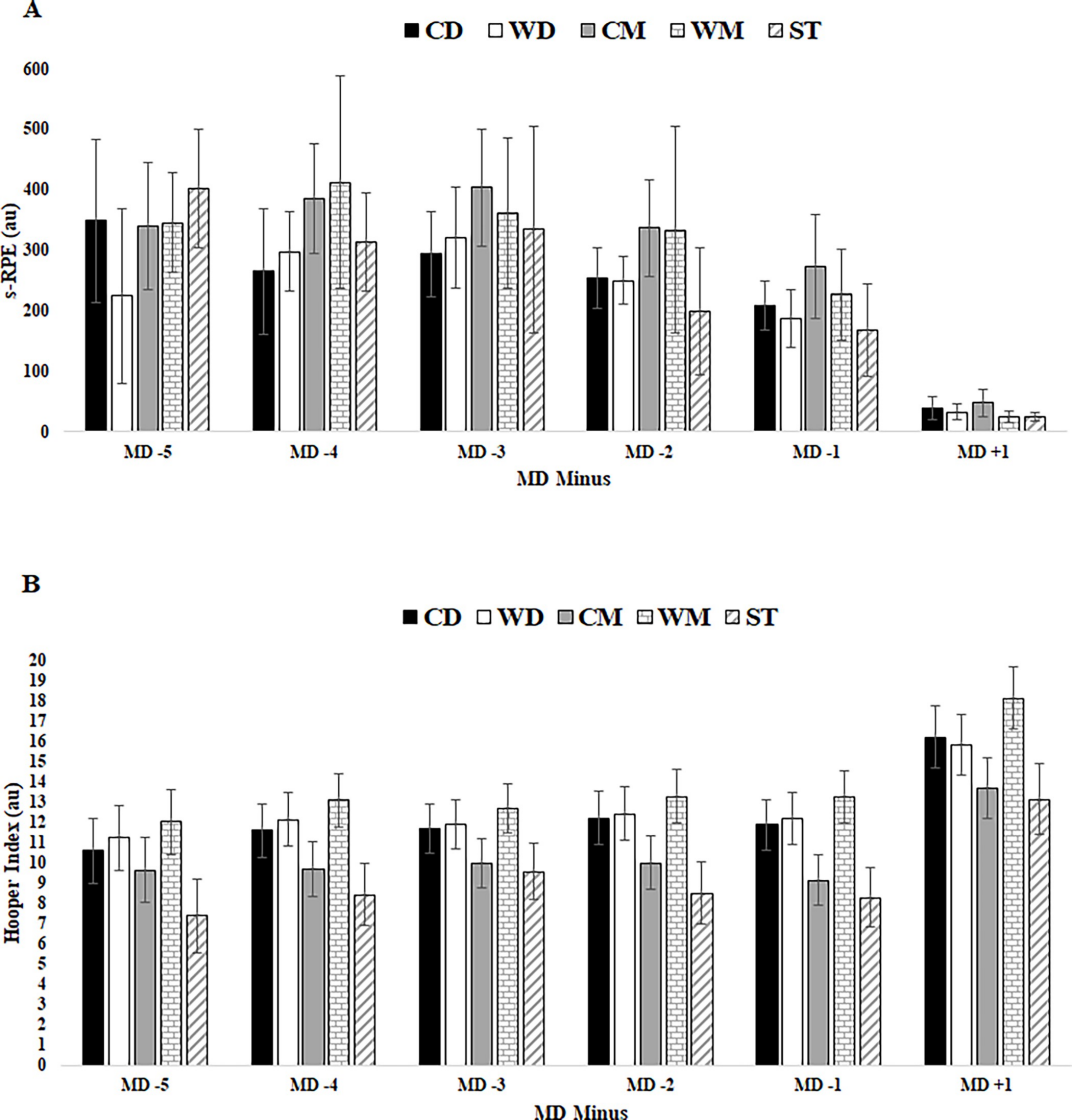
Internal TL data for s-RPE and HI in respect to days before a competitive match between player positions. Abbreviations: A) s-RPE; (B) HI; (CD), central defenders; (WD), wide defenders; (CM), central midfielders; (WM), wide midfielders; (ST), strikers. (a) denotes significant difference in CD versus WD, (b) denotes.
